# Paired Maternal and Neonatal Telomere Length at Birth: An Exploratory Study of Mother–Infant Dyads

**DOI:** 10.3390/healthcare14213649

**Published:** 2026-10-28

**Authors:** Maria Cezara Muresan, Adrian Ratiu, Cosmin Citu, Anca Mihaela Bina, Ahmed Abu-Awwad, Simona-Alina Abu-Awwad, Zoran Laurentiu Popa

**Affiliations:** 1 Department of Obstetrics and Gynecology, Faculty of Medicine, “Victor Babes” University of Medicine and Pharmacy, 300041 Timișoara, Romania; muresan.maria@umft.ro (M.C.M.); ratiu.adrian@umft.ro (A.R.); citu.ioan@umft.ro (C.C.); popa.zoran@umft.ro (Z.L.P.); 2 “Pius Brînzeu” Emergency Clinical County Hospital, Bld Liviu Rebreanu, No. 156, 300723 Timisoara, Romania; ahm.abuawwad@umft.ro; 3 Centre for Translational Research and Systems Medicine, “Victor Babes” University of Medicine and Pharmacy, 300041 Timisoara, Romania; 4 Department XV-Discipline of Orthopedics-Traumatology, “Victor Babes” University of Medicine and Pharmacy, 300041 Timisoara, Romania; 5 Research Center University Professor Doctor Teodor Sora, “Victor Babes” University of Medicine and Pharmacy, 300041 Timisoara, Romania

**Keywords:** maternal telomere length, neonatal telomere length, umbilical cord blood, mother–infant pairs, birth cohort, quantitative polymerase chain reaction

## Abstract

**Highlights:**

**What are the main findings?**

- In 150 mother–infant pairs, neonatal telomere length exceeded maternal telomere length in 83.3% of pairs and showed a moderate positive correlation with maternal telomere length.
- Maternal telomere length was positively associated with neonatal telomere length in the adjusted model. Clinical associations with maternal age and newborn sex were exploratory; only the unadjusted maternal-age association remained significant after Holm–Bonferroni correction.

**What are the implications of the main findings?**

- Maternal and neonatal telomere measurements are related at birth, but the variation between pairs limits what a maternal measurement can tell us about an individual newborn.
- Larger studies with repeated measurements are needed before telomere length can be considered for clinical prediction or screening.

**Abstract:**

Background/Objectives: Telomere length varies between individuals from birth, but the relationship between maternal and neonatal measurements within the same family remains incompletely understood. This study examined paired telomere length at delivery and explored associations with maternal and neonatal clinical characteristics. Methods: In this single-center observational study, maternal peripheral blood and neonatal umbilical cord blood were collected from 150 mother–infant pairs. Telomere length was measured by quantitative polymerase chain reaction. Paired measurements were compared using the Wilcoxon signed-rank test, and their association was assessed using Spearman’s correlation. Exploratory clinical associations were examined with unadjusted analyses and multivariable linear regression. Results: The median assay-derived estimate of total telomere length (TTL) was 1.29 Mb per diploid cell (IQR, 0.98–1.53) in neonatal samples and 0.94 Mb per diploid cell (IQR, 0.77–1.10) in maternal samples. Neonatal TTL estimates exceeded maternal estimates in 125 of 150 pairs (83.3%). The median within-pair difference was 0.35 Mb per diploid cell (IQR, 0.08–0.61; 95% CI, 0.27–0.43; *p* < 0.001). Maternal and neonatal TTL estimates were positively correlated (Spearman’s ρ = 0.479; 95% CI, 0.35–0.59; *p* < 0.001). In an exploratory adjusted model, maternal TTL was positively associated with neonatal TTL (β = 0.662 Mb per diploid cell per 1 Mb increase in maternal TTL; 95% CI, 0.453–0.871). Maternal age showed an inverse association with neonatal TTL (β = −0.013 Mb per diploid cell per year; 95% CI, −0.022 to −0.003), while female newborn sex showed a positive association (β = 0.128 Mb per diploid cell; 95% CI, 0.019–0.238). Prepregnancy BMI, gestational age, and active maternal smoking showed no clear adjusted associations. These clinical findings are exploratory: only the unadjusted maternal-age association remained statistically significant after Holm–Bonferroni correction across the 16 clinical tests; the sex-related associations did not. Conclusions: Maternal and neonatal telomere lengths were related at birth, although neonatal values varied substantially among pairs with similar maternal values. Larger studies are needed to confirm the secondary clinical associations and assess their potential clinical relevance.

## 1. Introduction

Telomeres protect chromosome ends and help maintain genomic stability during cell division. Their length varies between individuals, and these differences are already present at birth. The neonatal period is therefore a useful starting point for studying telomere biology, before years of postnatal exposures have accumulated. It is only a starting point, however. Longitudinal studies have shown that telomere length changes during childhood and even within the first year of life [1,2]. At the same time, measurements obtained at birth have been related to telomere length later in life [3] and, in one cohort, to blood pressure at ages four to six years [4]. These findings make the initial telomere profile worth studying, although they do not establish its value as a clinical prediction test.

Neonatal telomere length may reflect both inherited characteristics and the conditions in which pregnancy develops. In a large mother–offspring cohort, longer maternal telomere length was associated with longer newborn telomere length. Measures of maternal metabolic and nutritional health were also associated with newborn values [5]. Research into genetic variation and DNA methylation suggests that regulation of telomere length at birth involves several biological layers [6]. These influences are difficult to separate in an observational study. Even when maternal and newborn measurements are related, the association cannot show on its own how much is inherited and how much reflects their shared environment during pregnancy.

Studies of prenatal exposures illustrate why a single explanation is unlikely. Maternal urinary selenium concentrations have been associated with newborn telomere length [7]. Another cohort found associations between maternal fatty acid profiles and telomere length in cord blood, with different patterns observed in placental samples [8]. Gestational weight gain has also been examined in relation to telomere measurements [9], and one study reported shorter newborn leukocyte telomere length when maternal weight gain was below recommended levels [10]. Maternal psychosocial stress [11] and blood pressure during pregnancy [12] have been investigated as well. These studies used different populations, samples and methods. Their results support continued investigation, but they should not be read as evidence that any one maternal characteristic determines an individual newborn’s telomere length.

Paired maternal and neonatal measurements provide a way to examine the relationship within the same family. For example, a study that measured maternal and cord blood telomere length at delivery found different patterns of association with prenatal air pollution exposure in the two sample types [13]. Gestational age and birth weight may also need consideration when interpreting neonatal measurements, although findings from a study of preterm infants cannot simply be extended to all births [14]. More directly, a recent study of mothers and their preterm or term newborns reported a modest positive correlation between their telomere lengths [15]. Taken together, this work shows the value of studying the pair while also considering the population and biological material being analyzed.

For an exploratory clinical study, the first question is straightforward: how do maternal and neonatal telomere measurements relate within individual pairs? Describing their distributions and the variation between dyads is useful before asking whether telomere length might eventually contribute to personalised care. A small study cannot establish a predictive biomarker or explain the mechanisms behind an observed association. It can, however, provide a carefully described clinical dataset and identify questions for larger cohorts.

In the present study, we examined paired maternal and neonatal telomere length measurements obtained around birth. We aimed to describe the measurements in each group, explore their relationship within mother–infant dyads, and assess associations with the available maternal and neonatal clinical characteristics. Our focus is the paired profile at birth and its interpretation in an obstetric setting.

## 2. Materials and Methods

### 2.1. Study Design and Participants

This exploratory observational study was conducted at the “Pius Brînzeu” County Emergency Clinical Hospital in Timișoara, Romania. Mothers and newborns were recruited between January 2022 and December 2025 through the hospital’s Obstetrics and Gynecology Departments I and II and Neonatology Department. The study examined telomere length in paired maternal blood and neonatal umbilical cord blood samples collected around the time of birth. This was a prospective observational study. All eligible women presenting during the recruitment period were approached consecutively. Only singleton pregnancies were included; no multiple gestations were included. No mother contributed more than one infant or more than one pregnancy to the study.

Mother–infant dyads were eligible for the paired analysis if: (1) the mother delivered at the study hospital during the recruitment period; (2) a maternal blood sample and an umbilical cord blood sample were obtained for telomere length analysis; (3) both samples produced conclusive numerical results; (4) the maternal and neonatal samples could be reliably linked to the same delivery; and (5) the required consent for participation and sample collection was documented. Only singleton pregnancies were included, and no mother contributed more than one infant.

Of the 167 mother–infant dyads assessed, 17 were excluded for the following reasons: five participants withdrew consent; six dyads could not be included because the maternal and neonatal samples had been mixed up and could not be reliably matched; two dyads lacked a neonatal sample; three had incomplete consent documentation; and one had essential clinical data missing. The remaining 150 dyads were included in the paired analysis. All included dyads had conclusive maternal and neonatal telomere-length results, documented consent, reliable sample linkage, and the clinical data required for the reported analyses.

Maternal age, smoking status, medical conditions and mode of delivery were not used as exclusion criteria for this analysis. This approach allowed the paired measurements to be examined across the clinical characteristics represented in the available cohort.

Although the present study included an independently recruited cohort of 150 mother-infant dyads and examined paired, aternal and neonatal telomere-length measuremenr.

### 2.2. Clinical Data

Maternal and neonatal information was obtained from the obstetric and neonatal records. Available maternal variables included age, smoking status, recorded conditions and laboratory findings. Prepregnancy weight was self-reported by the participants, and height was obtained from the medical records. BMI was calculated as weight in kilograms divided by height in meters squared. Maternal smoking status was self-reported at the first antenatal visit and recorded as a binary variable (current active smoking: yes/no). Smoking status was not biochemically verified. Maternal conditions were obtained from the obstetric records and included chronic hypertension, gestational hypertension, preeclampsia, pre-existing diabetes, gestational diabetes, thyroid disease, anemia, and bronchial asthma. Obstetric data included pregnancy plurality and mode of delivery. Neonatal data included sex, birth weight, gestational age at delivery, and Apgar scores at 1 and 5 min. The laboratory dataset used identifiers ending in “M” for maternal samples and “C” for the corresponding neonatal samples. These identifiers were used to assemble the paired dataset; identifiable clinical information was not included in the analytical file. Parity was recorded before the index delivery. Women with no previous births were classified as nulliparous and were therefore primiparous at the index delivery; women with one or more previous births were classified as multiparous. In the sensitivity analyses, parity was entered as a binary variable (multiparous vs. primiparous at the index delivery), with primiparous women as the reference category.

The primary analysis included 150 mother–infant dyads (150 mothers and 150 newborns), each with conclusive maternal and neonatal telomere length measurements. All 150 dyads had complete data for the clinical variables reported in Table 1, Table 2, Table 3, Table 4 and Table 5; no observations were missing for these variables within the analytical cohort.

### 2.3. Sample Collection and Telomere Length Measurement

Maternal peripheral venous blood was collected at delivery in 2-mL tubes containing ethylenediaminetetraacetic acid (EDTA), and neonatal umbilical cord blood was collected in EDTA tubes at birth. Samples were processed on the day of collection. After centrifugation, the leukocyte-rich buffy-coat fraction was separated and stored at −80 °C for up to 2 months before DNA extraction. Genomic DNA was extracted using the Maxwell^®^ RSC Buffy Coat DNA Kit on the Maxwell^®^ RSC 48 Instrument (Promega Corporation, Madison, WI, USA). Extracted DNA was stored at −80 °C for up to 10 months before qPCR analysis. No freeze–thaw cycles occurred before analysis.

For clarity, maternal telomere length refers to the assay-derived estimate from leukocytes isolated from maternal peripheral venous blood, whereas neonatal telomere length refers to the estimate from leukocytes isolated from umbilical cord blood collected at birth. In this study, “neonatal telomere length” and “newborn telomere length” refer to the same assay-derived estimate from leukocytes isolated from umbilical cord blood collected at birth; “newborn” describes the infant at birth. “Fetal telomere length” is retained only when reflecting terminology used in cited studies; no separate fetal sample was measured in the present study.

Telomere length was measured using the Absolute Human Telomere Length Quantification qPCR Assay Kit (ScienCell Research Laboratories, Carlsbad, CA, USA; cat. no. 8918) on an ABI 7900HT Real-Time PCR System (Thermo Fisher Scientific, Waltham, MA, USA). DNA concentration was measured fluorometrically, and samples were diluted to 2 ng/µL. The median A260/A280 ratio was 1.82 (range, 1.76–1.89), and the median A260/A230 ratio was 2.08 (range, 1.90–2.24). Separate reactions were performed for the telomere sequence (TEL) and the single-copy reference (SCR). Each reaction had a final volume of 20 µL and contained 1 µL of DNA template, 2 µL of the corresponding primer stock, 10 µL of 2× qPCR master mix, and 7 µL of nuclease-free water, giving 2 ng of DNA per reaction. The manufacturer-recommended DNA template concentration is 0.5–5 ng/µL; samples were analyzed at 2 ng/µL. Each sample was analyzed in triplicate. The cycling program consisted of an initial denaturation at 95 °C for 10 min, followed by 32 cycles of 95 °C for 20 s, 52 °C for 20 s, and 72 °C for 45 s.

Technical triplicates were considered acceptable when the standard deviation (SD) of Cq values was ≤0.5. Samples with an initial SD > 0.5 were repeated, and all repeat assays met the acceptance criterion. The kit-provided reference genomic DNA was used as the calibrator and was included on each plate; an NTC was also included on each plate, with no amplification observed. Technical triplicates for TEL and SCR reactions were grouped together. Paired maternal and neonatal samples were placed in the same area of the plate, while the calibrator and NTC were placed in predefined positions. Telomere and SCR amplification efficiencies were 98% (R^2^ = 0.996) and 97% (R^2^ = 0.995), respectively. Intra-assay and inter-assay coefficients of variation, measured using the same control DNA sample, were 3.8% and 6.2%, respectively. The assay-derived estimate of total telomere length (TTL) for each sample was calculated relative to the kit-provided reference genomic DNA using the comparative ΔΔCq method. ΔCq_TEL was calculated as Cq_TEL,target − Cq_TEL,calibrator, and ΔCq_SCR as Cq_SCR,target − Cq_SCR,calibrator; ΔΔCq was calculated as ΔCq_TEL − ΔCq_SCR. TTL_target (Mb per diploid cell) was calculated as TTL_calibrator × 2^−ΔΔCq. The assigned telomere length of the calibrator was 1.23 ± 0.09 Mb per diploid cell [16–17]. The calibrator varied by no more than 0.25 Cq between plates. Plate effects were assessed by one-way ANOVA, which did not show a statistically significant difference between plates (*p* > 0.05). Samples were coded, and laboratory personnel were blinded to clinical information. The calibration curves and amplification efficiencies for the TEL and SCR assays were determined according to the manufacturer’s instructions for the kit.

### 2.4. Outcomes and Statistical Analysis

The primary outcome was the association between maternal and neonatal assay-derived estimates of total telomere length (TTL) within mother–infant dyads. Secondary descriptive outcomes were the distribution of maternal and neonatal TTL estimates and the magnitude and direction of the within-pair difference (neonatal minus maternal). TTL estimates and within-pair differences are expressed in megabases per diploid cell (Mb per diploid cell). Associations between neonatal TTL estimates and available maternal and neonatal clinical characteristics were considered exploratory.

No formal a priori sample-size calculation was performed for the primary maternal–neonatal correlation. The sample size was determined by feasibility and the number of eligible, consecutively recruited dyads available during the prespecified recruitment period from January 2022 to December 2025. Of the 167 dyads assessed, 150 met the inclusion criteria and had analyzable paired measurements. Accordingly, the study was not specifically designed to detect small correlations or to provide precise estimates for exploratory subgroup analyses.

Continuous variables were summarized as mean ± standard deviation or median and interquartile range, according to their distributions. Categorical variables were presented as counts and percentages. The analyses of maternal and neonatal telomere length used the 150 dyads with conclusive measurements in both samples; all analyses involving the clinical characteristics reported in this manuscript included the same 150 dyads. No formal a priori sample-size or power calculation was performed. The final sample size was determined by the number of eligible mother–infant dyads recruited during the study period with conclusive paired maternal and neonatal telomere-length measurements. Associations with clinical characteristics were considered exploratory. Holm–Bonferroni adjustment was applied across the 16 exploratory tests of clinical characteristics reported in Table 3, Table 4 and Table 5. The primary paired maternal–neonatal telomere-length association was considered separately. Both unadjusted and adjusted *p* values are reported.

Spearman’s rank correlation coefficient (ρ) was used to assess the association between maternal and neonatal telomere length. The Wilcoxon signed-rank test was used to compare the paired measurements. The within-pair difference was calculated as neonatal minus maternal telomere length. Exploratory associations of neonatal telomere length with maternal age, prepregnancy body mass index (BMI), gestational age, and birth weight were assessed using Spearman’s correlation. For comparisons of neonatal telomere length between newborn sex and maternal smoking groups, normality within each group was assessed using Q–Q plots and the Shapiro–Wilk test; no important departures from normality were identified. Welch’s independent-samples *t*-test was used for these comparisons, as it does not assume equal variances between groups. The corresponding differences between group means were expressed in Mb per diploid cell [17]. Two-sided 95% CIs for Spearman’s ρ were obtained using the approximation implemented in GraphPad Prism version 6. The 95% CI for the median paired difference was calculated using the Hodges–Lehmann method as part of the Wilcoxon matched-pairs signed-rank analysis in GraphPad Prism version 6. For comparisons by newborn sex and maternal smoking, 95% CIs for unadjusted mean differences were calculated using Welch’s unequal-variance independent-samples *t*-test.

Two multivariable linear regression models were fitted. In the first model, neonatal telomere length was the dependent variable; maternal telomere length, maternal age, prepregnancy BMI, gestational age, newborn sex, and active maternal smoking were included as independent variables. In the second model, the neonatal-minus-maternal telomere length difference was the dependent variable; maternal age, prepregnancy BMI, gestational age, newborn sex, and active maternal smoking were included as independent variables. To limit potential part–whole mathematical coupling, maternal telomere length was not included as a predictor in the model in which the outcome was calculated as neonatal minus maternal telomere length. The models were exploratory, and covariates were not formally prespecified in a statistical analysis plan. Covariates in the primary models were selected on the basis of clinical and biological relevance and data availability, rather than automated or significance-based selection. The model with neonatal telomere length as the outcome also served as a sensitivity analysis addressing the limitations of the neonatal-minus-maternal difference score. Birth weight was not included alongside gestational age because of their conceptual overlap. Apgar score was not included in the primary models because it reflects immediate neonatal adaptation and was not considered an a priori confounder of telomere length. To examine the influence of other available clinical characteristics, Apgar score at 5 min (0–10), parity (multiparous vs. primiparous), mode of delivery (cesarean vs. vaginal), and a binary maternal comorbidity variable (coded as present if at least one of the following was documented: chronic or gestational hypertension, preeclampsia, pre-existing or gestational diabetes, thyroid disease, anemia, or bronchial asthma; coded as absent if none was documented) were each added separately to the corresponding primary model in exploratory sensitivity analyses. All 150 dyads were included in each primary and sensitivity model. Regression coefficients (B) are reported as unstandardised estimates with 95% confidence intervals. Model assumptions were assessed by reviewing residual diagnostics for linearity, homoscedasticity, residual normality, and influential observations. Multicollinearity was assessed using variance-inflation factors (VIFs) and tolerance. All tests were two-sided, and *p* < 0.05 was considered nominally significant. Multiplicity adjustment was applied to the exploratory clinical association analyses using the Holm–Bonferroni method. Both nominal and multiplicity-adjusted *p* values are reported. These analyses were considered hypothesis-generating, and interpretation focused on effect estimates, 95% confidence intervals, and the results of multiplicity adjustment.

Descriptive analyses, the paired comparison, Spearman correlations and graphical presentation were performed using GraphPad Prism, version 5 (GraphPad Software, San Diego, CA, USA). Multivariable linear regression was performed using IBM SPSS Statistics software Version 29 (IBM Corp., Armonk, NY, USA). The completed STROBE and MIQE 2.0 reporting checklists are provided as Supplementary Files S1 and S2, respectively.

### 2.5. Ethical Considerations

The study was approved by the Ethics Committee of the “Pius Brînzeu” County Emergency Clinical Hospital, Timișoara, Romania (approval No. 225, dated 2 September 2021). The approval was valid for 5 years and covered the collection and telomere-length testing of paired maternal peripheral blood and neonatal umbilical cord blood samples. Recruitment took place between January 2022 and December 2025, within the approval period. Written informed consent was obtained from all participating mothers. Each mother signed a separate section for her own participation and a separate section consenting to the collection of her newborn’s sample. The consent form stated that study data would remain anonymized in the hospital database and could be reused for 10 years from the date of sample collection. The biological specimens were analyzed for the present study and were not reused in separate studies.

## 3. Results

### 3.1. Study Population

**Figure 1:**
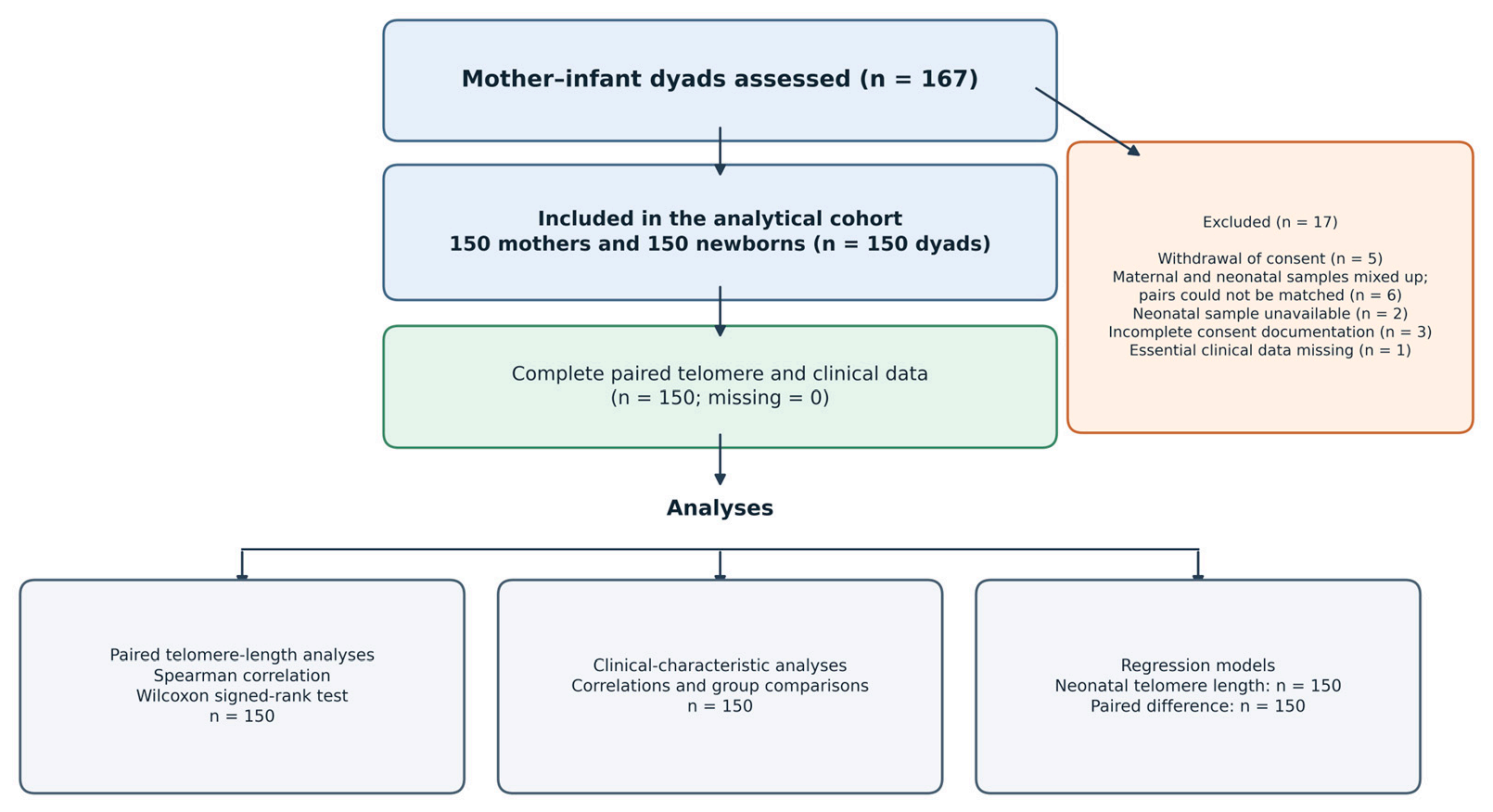
Flow diagram of mother–infant dyad selection. Of 167 dyads assessed for eligibility, 17 were excluded for the reasons shown, and 150 were included in the paired analysis.

Of the 167 mother–infant dyads assessed for eligibility, 17 were excluded: five participants withdrew consent, six dyads could not be reliably established because maternal and neonatal samples had been mixed up, two lacked a neonatal sample, three had incomplete consent documentation, and one had essential clinical data missing. The remaining 150 dyads were included in the analyses. All 150 had conclusive maternal and neonatal telomere-length results and complete data for the variables reported in this study (Figure 1).

The mean maternal age was 31.4 ± 5.3 years, and the mean prepregnancy body mass index (BMI) was 24.7 ± 4.3 kg/m^2^. Fifty-six women (37.3%) were nulliparous, and 26 (17.3%) reported active smoking. Delivery occurred at a mean gestational age of 38.6 ± 1.6 weeks; 16 infants (10.7%) were born before 37 weeks. Seventy-six newborns (50.7%) were female, and the mean birth weight was 3198 ± 499 g. Recorded maternal conditions included chronic hypertension in 2 women (1.3%), gestational hypertension in 11 (7.3%), preeclampsia in 1 (0.7%), pre-existing diabetes in 1 (0.7%), gestational diabetes in 7 (4.7%), thyroid disease in 3 (2.0%), anemia in 17 (11.3%), and bronchial asthma in 2 (1.3%). All pregnancies were singleton. Vaginal birth occurred in 111 cases (74.0%), and 39 deliveries (26.0%) were by cesarean section. The mean Apgar scores were 8.2 ± 0.7 at 1 min and 8.9 ± 0.6 at 5 min (Table 1).

### 3.2. Assay-Derived Estimates of Maternal and Neonatal Total Telomere Length

**Figure 2:**
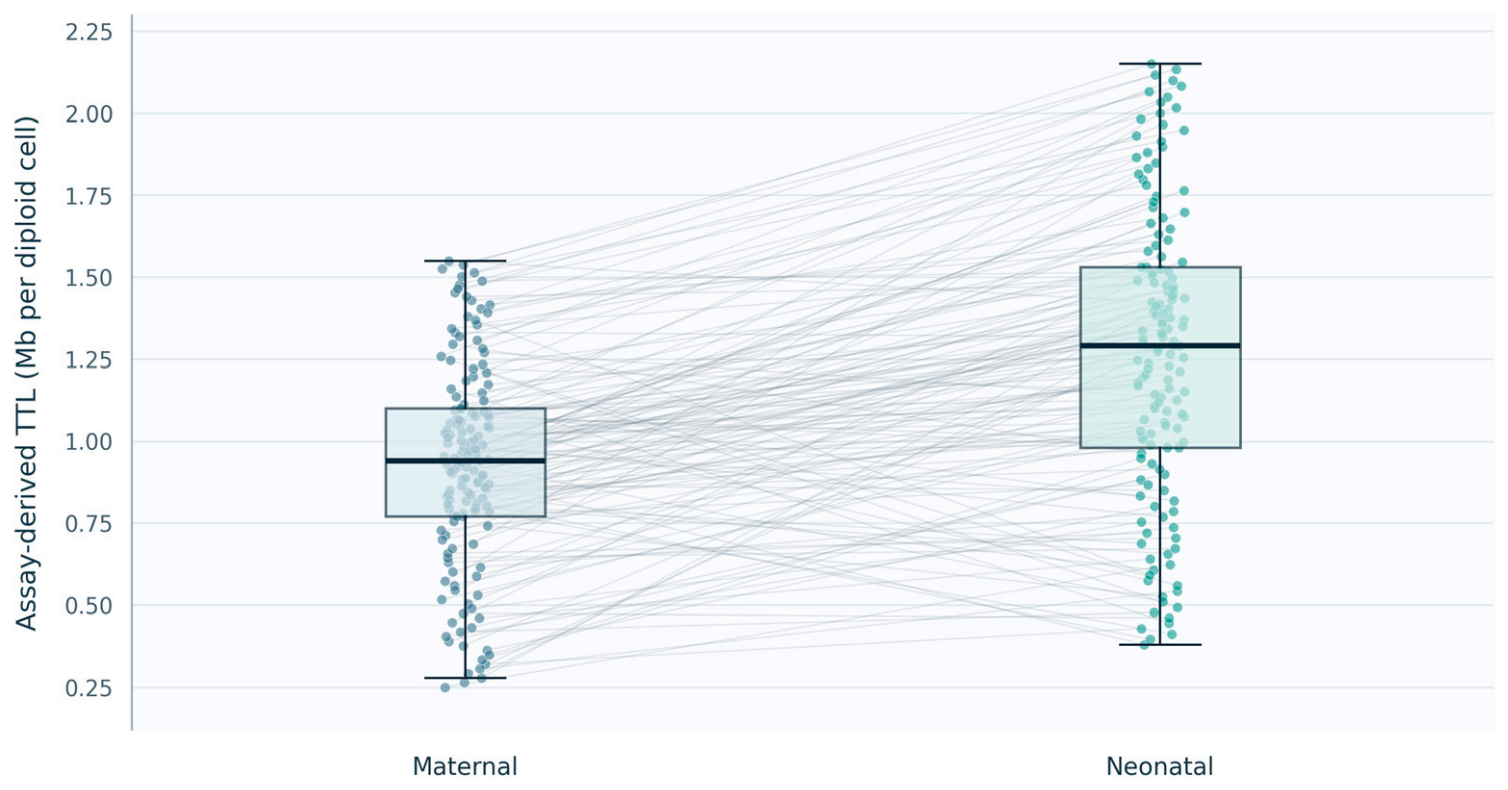
Paired plot of maternal and neonatal assay-derived estimates of total telomere length (TTL) at birth. Each point represents an observed measurement, and connecting lines link the maternal and neonatal measurements from the same mother–infant dyad (*n* = 150). Boxes indicate the interquartile range, horizontal lines indicate the median, and whiskers extend to 1.5 times the interquartile range.

The median neonatal assay-derived TTL estimate was 1.29 Mb per diploid cell (IQR, 0.98–1.53), compared with 0.94 Mb per diploid cell (IQR, 0.77–1.10) for maternal samples. The median within-pair difference (neonatal minus maternal) was 0.35 Mb per diploid cell (IQR, 0.08–0.61; 95% CI, 0.27–0.43). Neonatal TTL estimates exceeded the corresponding maternal estimates in 125 of 150 dyads (83.3%). The paired difference was statistically significant (Wilcoxon signed-rank test, *p* < 0.001; Table 2 and Figure 2).

**Figure 3:**
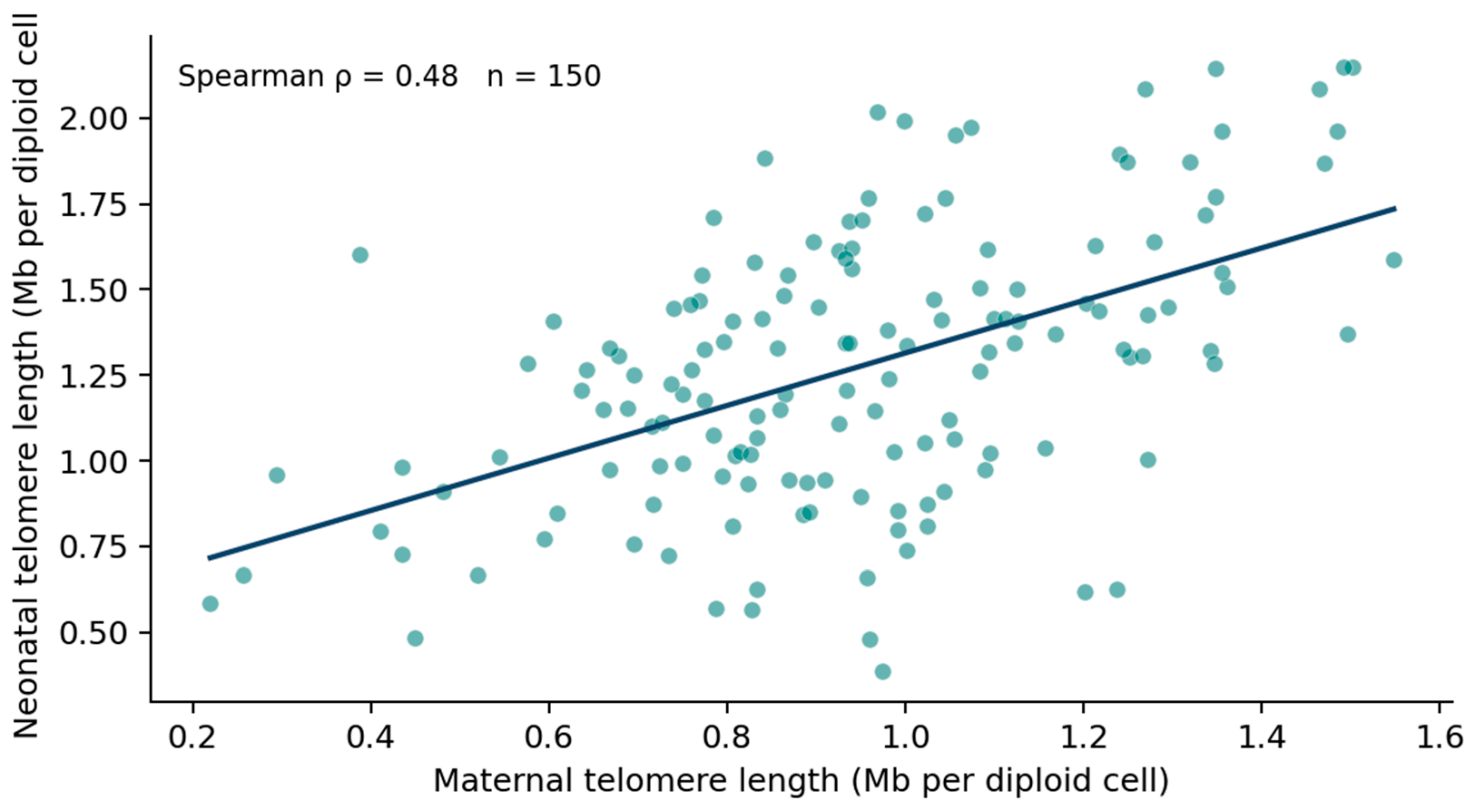
Association between maternal and neonatal assay-derived estimates of total telomere length (TTL) in mother–infant dyads. Each point represents one of the 150 paired measurements. The solid line represents an ordinary least-squares linear fit and is shown for descriptive visualization only. The association was assessed using Spearman’s rank correlation coefficient (ρ = 0.479; *p* < 0.001). TTL estimates are expressed in megabases per diploid cell (Mb per diploid cell).

Across dyads, maternal and neonatal measurements showed a positive association (Spearman’s ρ = 0.479, 95% CI [0.35–0.59], *p* < 0.001; Table 2 and Figure 3). The scatterplot also shows variation in neonatal measurements among dyads with similar maternal values.

### 3.3. Exploratory Associations with Clinical Characteristics

In the unadjusted analyses, neonatal assay-derived estimates of total telomere length (TTL) were inversely correlated with maternal age (ρ = −0.320, *p* < 0.001) and prepregnancy BMI (ρ = −0.180, *p* = 0.028). Correlations with gestational age (ρ = 0.101, *p* = 0.218) and birth weight (ρ = 0.119, *p* = 0.147) were smaller and did not reach the nominal significance threshold. Female newborns had a median neonatal TTL of 1.34 Mb per diploid cell (IQR, 1.02–1.58), compared with 1.21 Mb per diploid cell (IQR, 0.91–1.47) in male newborns. The unadjusted mean difference (female minus male) was 0.128 Mb per diploid cell (95% CI, 0.016–0.240; *p* = 0.025). Among newborns of active smokers, the median neonatal TTL was 1.34 Mb per diploid cell (IQR, 0.97–1.66), compared with 1.28 Mb per diploid cell (IQR, 0.98–1.52) among newborns of non-smokers. The unadjusted mean difference (active smoking minus no active smoking) was 0.056 Mb per diploid cell (95% CI, −0.175 to 0.287; *p* = 0.632; Table 3).

All six analyses in this table included the same 150 dyads; no values were missing for the listed characteristics. The comparison by newborn sex included 76 female and 74 male newborns. The comparison by maternal smoking included 26 active smokers and 124 non-smokers. The clinical associations reported in this section were exploratory and hypothesis-generating. Both nominal and Holm–Bonferroni-adjusted *p* values are presented to account for multiple comparisons. Interpretation of the findings emphasizes effect estimates, confidence intervals, and the results of multiplicity adjustment.

### 3.4. Multivariable Analyses

**Figure 4:**
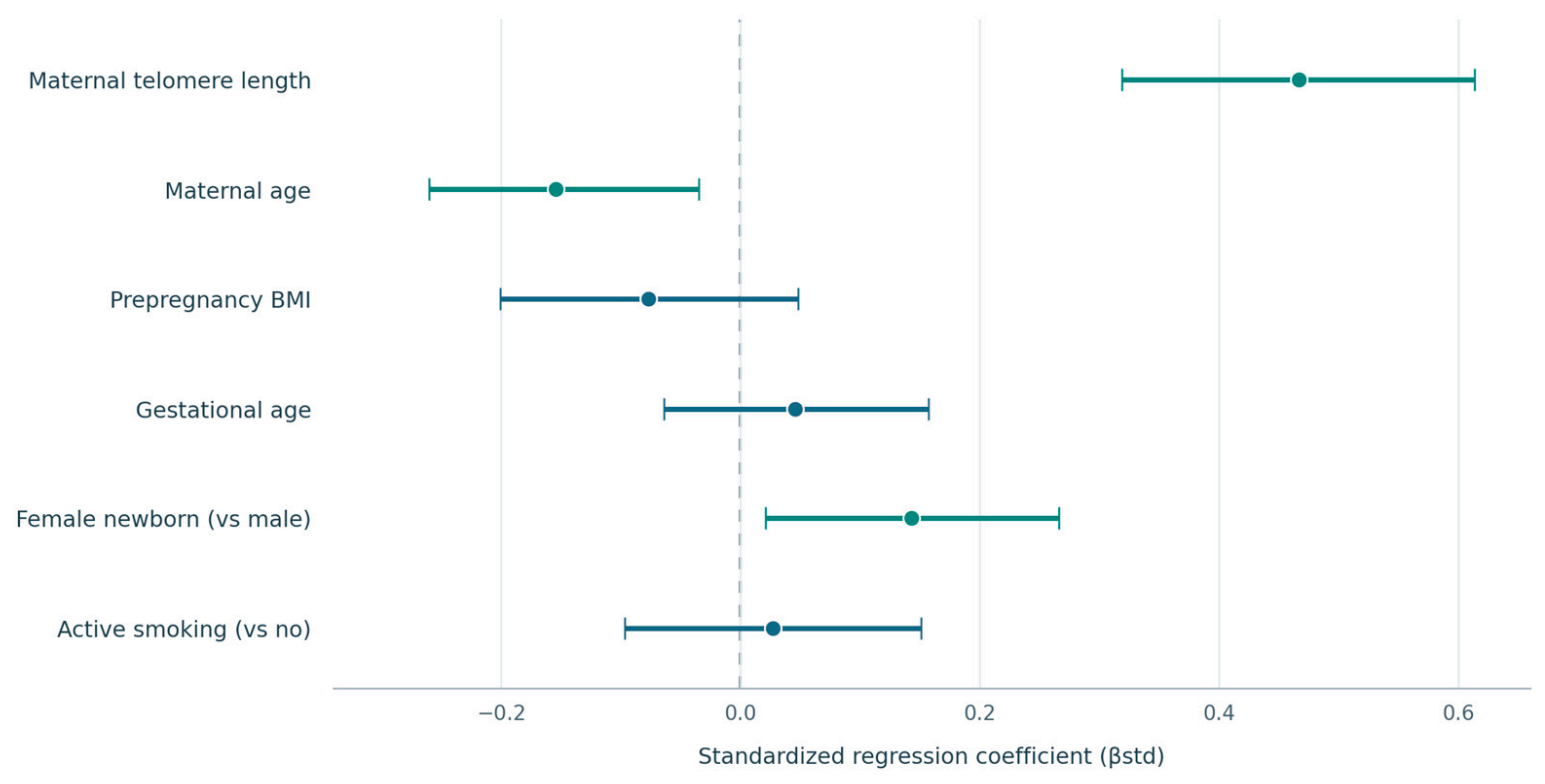
Standardized regression coefficients for the adjusted associations of maternal telomere length, maternal age, prepregnancy body mass index, gestational age, newborn sex, and active maternal smoking with neonatal total telomere length (*n* = 150). Points represent standardized coefficients (βstd), and horizontal bars represent 95% confidence intervals. The dashed vertical line indicates the null value. Teal intervals exclude zero; blue intervals include zero.

In the model with neonatal telomere length as the outcome, maternal telomere length remained positively associated with the neonatal measurement after adjustment for maternal age, prepregnancy BMI, gestational age, newborn sex, and active maternal smoking (β = 0.662 Mb per diploid cell per 1 Mb increase in maternal telomere length; 95% confidence interval [CI], 0.453–0.871; *p* < 0.001). Maternal age was inversely associated with neonatal telomere length (β = −0.013 Mb per diploid cell per year; 95% CI, −0.022 to −0.003; *p* = 0.008), while female sex showed a positive association (β = 0.128 Mb per diploid cell; 95% CI, 0.019–0.238; *p* = 0.021). The estimates for BMI, gestational age, and smoking had 95% CIs that crossed zero (Table 4 and Figure 4). The model included 150 dyads and had an intercept of 0.70 Mb per diploid cell, R^2^ = 0.32, adjusted R^2^ = 0.29, and RMSE = 0.34 Mb per diploid cell. This model provides a complementary analysis that does not use the neonatal-minus-maternal difference score as the outcome.

**Figure 5:**
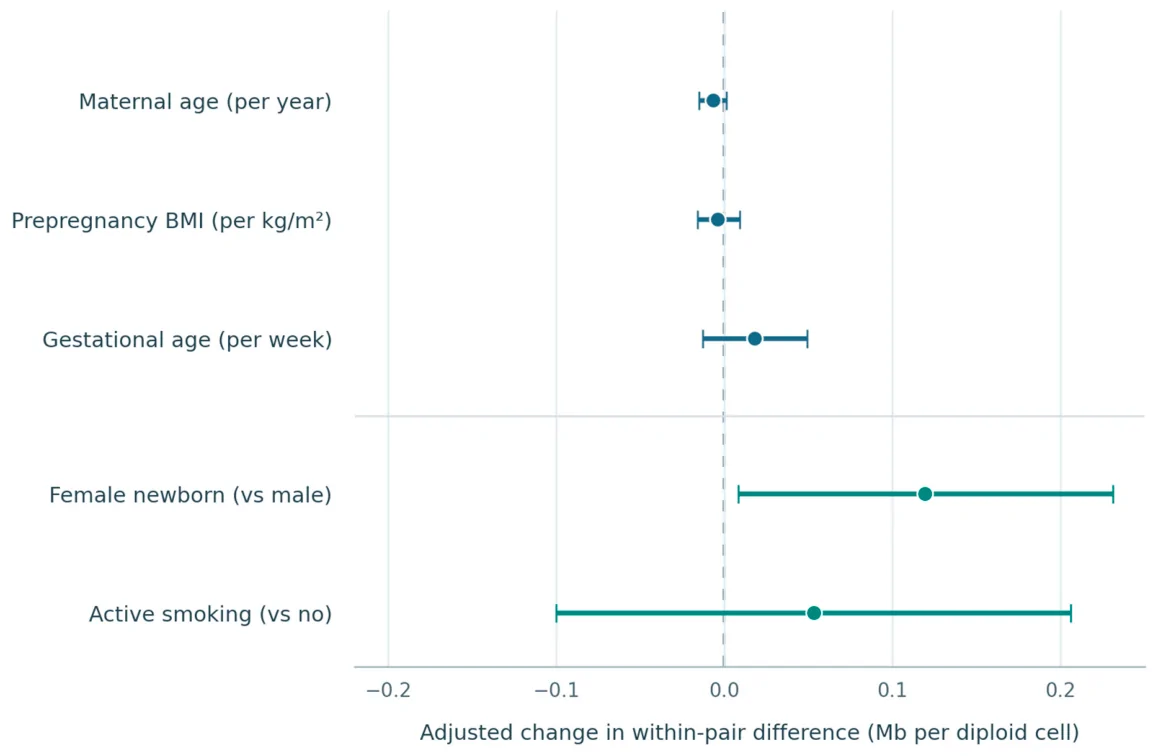
Adjusted associations of maternal age, prepregnancy body mass index, gestational age, newborn sex, and active maternal smoking with the within-pair difference in telomere length (neonatal minus maternal; Mb per diploid cell; *n* = 150). Points represent unstandardized regression coefficients, and horizontal bars represent 95% confidence intervals. Continuous predictors are shown separately from categorical comparisons; units are specified for continuous predictors. The dashed vertical line indicates the null value.

A second model used the within-pair difference between the neonatal and maternal assay-derived estimates of total telomere length (TTL), calculated as the neonatal TTL estimate minus the maternal TTL estimate, as the outcome. Female newborn sex was associated with a larger difference (β = 0.119 Mb per diploid cell; 95% CI, 0.008–0.231; *p* = 0.036). The 95% CIs for maternal age, BMI, gestational age, and smoking all included zero (Table 5 and Figure 5). This model addresses variation in the paired difference and is distinct from the model for neonatal telomere length. The sensitivity analyses are presented in Table 6. The model included 150 dyads and had an intercept of −0.10 Mb per diploid cell, R^2^ = 0.10, adjusted R^2^ = 0.07, and RMSE = 0.35 Mb per diploid cell. Across both models, diagnostic review found approximately linear relationships, no marked heteroscedasticity, approximately normally distributed residuals, no unduly influential observations, and low multicollinearity. The maximum VIF was 2.2 and the minimum tolerance was 0.45.

After Holm–Bonferroni adjustment across the 16 exploratory tests of clinical characteristics, only the maternal-age association reported in Table 3 remained statistically significant (Holm-adjusted *p* = 0.016). The associations with prepregnancy BMI and newborn sex in Table 3, and the sex-related findings in the multivariable models, did not remain statistically significant after adjustment. No other clinical-characteristic association remained statistically significant.

## 4. Discussion

In this cohort of 150 mother–infant dyads, maternal and neonatal assay-derived estimates of total telomere length (TTL) were related but far from interchangeable. The association was moderate, and the scatterplot shows a fairly wide range of neonatal TTL estimates at similar maternal TTL estimates. Neonatal TTL estimates were higher than maternal TTL estimates in most pairs. Previous paired studies likewise indicate that maternal measurements account for only part of the variation observed at birth [5,18]. These findings give us two complementary views of the same dataset: there is a measurable relationship within the dyad, while considerable variation remains at the level of the individual newborn. This distinction matters if paired measurements are to be used to understand early telomere biology rather than treated as a simple proxy for one another [19].

The positive association between maternal and neonatal TTL estimates is consistent with influences shared within families. Part of it could be genetic, and part could arise from conditions shared during pregnancy [5,20]. Our design cannot separate those possibilities. We did not measure paternal telomere length, inherited variants or the full range of prenatal exposures. A study of maternal inflammatory status and research on variation in telomere length across blood-cell types further illustrate why a single maternal measurement cannot capture every relevant influence [21,22]. The correlation between maternal and neonatal TTL estimates (ρ = 0.479) should not be interpreted as the proportion of the neonatal TTL estimate determined by the mother. It describes how the paired TTL estimates rank together in this sample. Some newborns had relatively high TTL estimates even when their mothers did not, and the reverse was also observed. That spread suggests we should look beyond a single maternal measurement in future work.

Importantly, the observed maternal–neonatal correlation reflects a statistical association at the cohort level and does not establish the ability to accurately predict telomere length in an individual newborn. Establishing clinical predictive utility would require the development and external validation of a dedicated prediction model, including formal assessment of predictive accuracy, calibration, and generalizability in independent populations. Such analyses were beyond the scope of the present exploratory study.

Beyond genetic and shared intrauterine influences, cellular mechanisms involved in metabolic regulation and senescence may also contribute to telomere biology. The mechanistic target of rapamycin (mTOR) pathway integrates nutrient availability, growth signals, and cellular energy status to regulate metabolism, protein synthesis, and cell growth. Experimental studies have demonstrated that amino acid-dependent mTORC1 signaling regulates pancreatic β-cell function and proliferation through mechanisms involving PDX1 [23,24]. Telomere dysfunction can activate DNA damage responses and cellular senescence, while metabolic stress, mitochondrial dysfunction, and altered growth signaling may influence the cellular environment in which telomere maintenance occurs. These processes are relevant to broader mechanisms of cellular aging and tissue homeostasis [25]. However, the mechanistic evidence cited here does not directly establish a relationship between mTOR activity and maternal–neonatal telomere length. In the present study, mTOR activity, telomerase expression, mitochondrial function, and markers of cellular senescence were not assessed. Consequently, the observed associations cannot be attributed to specific molecular pathways, and further mechanistic investigations are warranted.

Neonatal assay-derived estimates of total telomere length (TTL) exceeded maternal estimates in 83.3% of pairs, with a median within-pair difference of 0.35 Mb per diploid cell. This pattern requires cautious interpretation. The samples came from different blood sources: maternal peripheral blood and neonatal cord blood. Although both were analyzed using the same assay and are reported on the same analytical scale, their leukocyte populations may differ in composition. Because leukocyte subsets were not measured or adjusted for, an assay applied to mixed white blood cells may reflect these compositional differences as well as differences in telomere length within cell types [22,26]. The observed difference should therefore not be interpreted as direct telomere loss between birth and adulthood, nor does it establish the later-life TTL estimate for any individual newborn. Longitudinal studies have followed telomere length from birth into childhood, but assessing change requires repeated measurements in the same individuals [1,3]. Paired sampling in the present study describes the relationship between maternal and neonatal TTL estimates at delivery. Because the difference score is calculated from two assay measurements, measurement error in either measurement may affect the resulting contrast. Because maternal telomere length is a component of the difference score, including it as a predictor would create part–whole mathematical coupling; it was therefore excluded from that model, and findings from the difference-score analysis are interpreted descriptively and cautiously.

Maternal age was inversely associated with the neonatal assay-derived TTL estimate in the unadjusted analysis and remained associated after adjustment for the maternal TTL estimate and the other recorded characteristics. The adjusted coefficient was small on the scale of a single year, and its clinical meaning is not obvious from these data alone. Still, the fact that the association persisted alongside maternal telomere length makes it worth examining in a larger cohort. Age may stand in for several factors that we did not measure, including reproductive history, cumulative exposures and aspects of maternal health. Earlier studies from our center examined maternal telomere length in relation to neonatal parameters and maternal cardiovascular risk, respectively, but addressed different questions from the paired maternal–neonatal telomere analysis presented here [27,28]. We cannot tell from this analysis which unmeasured factors, if any, account for the observed association with maternal age. The estimate should be read as a relationship within this cohort, not as evidence that a change in maternal age would itself change neonatal telomere length.

Prepregnancy BMI showed a weaker inverse correlation with the neonatal assay-derived TTL estimate before adjustment, but its confidence interval crossed zero in the multivariable model. This change could reflect overlap with maternal age, maternal telomere length or other measured characteristics; it could also reflect limited precision. A systematic review and meta-analysis found an association between maternal BMI and offspring telomere length across other studies, although that finding does not determine what should be observed in this cohort [29]. We would not infer from the adjusted result that maternal metabolic status is irrelevant. BMI is a broad measure, and the present dataset does not describe metabolic health in enough detail to test that question. Likewise, we found no clear association of neonatal telomere length with gestational age or birth weight. Research including both term and preterm neonates has examined these relationships in different clinical samples [15]. Only 16 births were preterm in our cohort, so the study had limited opportunity to examine the full range of gestational maturity. A non-significant result here should not be treated as proof that these features have no relationship with telomere length.

Female newborns had higher assay-derived TTL estimates than male newborns in the exploratory comparison and in the adjusted model. Female sex was also associated with a larger neonatal-minus-maternal difference in the second model. These estimates point in the same direction, although they do not answer the same question. The second outcome depends on both measurements in the pair, and it should not be taken as a separate replication of the first result. Other birth-cohort studies have reported that associations between prenatal exposures and neonatal telomere length can differ by infant sex [12,30]. The differences in our cohort were modest and their confidence intervals were relatively close to zero. After Holm–Bonferroni adjustment for multiple comparisons, the sex-related associations did not remain statistically significant. These findings should therefore be interpreted as exploratory and hypothesis-generating rather than confirmatory. Effect estimates and 95% confidence intervals should guide interpretation, and independent studies are needed to confirm these observations.

Active maternal smoking was not clearly associated with either the neonatal TTL estimate or the within-pair difference between neonatal and maternal TTL estimates in the models presented here. This finding should be considered alongside evidence linking prenatal tobacco exposure to newborn telomere length or to telomere trajectories in infancy [31,32]. Only 26 women were recorded as active smokers, and smoking was handled as a yes-or-no characteristic. This leaves little information about duration, intensity, exposure earlier in pregnancy or passive smoking. Passive exposure has also been examined in relation to newborn telomere length [33]. Maternal psychosocial and obstetric characteristics may add further variation that the present dataset does not capture fully [34]. The wide confidence intervals are compatible with more than one underlying effect, so the result cannot rule out a relationship. These models were exploratory and do not establish predictive utility or a clinical threshold.

Strengths, Limitations and Future Directions

The main strength of this study is the linkage of maternal and cord blood measurements in 150 dyads, with sampling around delivery. The paired design lets us describe the relationship between measurements and the direction of the difference within each family. We also considered several clinical characteristics. These features make the results more informative than unrelated distributions of maternal and neonatal samples.

There are important limitations. This was a single-center observational cohort. Seventeen of the 167 dyads assessed were excluded for the reasons reported in Section 3.1 and Figure 1. The six dyads with uncertain maternal–neonatal sample linkage and the other exclusions may have affected the composition of the analyzed cohort; therefore, some selection bias cannot be ruled out. Maternal peripheral blood and neonatal umbilical cord blood differ in leukocyte composition, and telomere length varies across leukocyte subsets. Because DNA was extracted from buffy-coat leukocytes and leukocyte-subset proportions were not measured in this study, the qPCR results represent bulk-leukocyte telomere-length estimates. Therefore, the higher neonatal measurements may partly reflect differences in leukocyte composition rather than longer telomeres within comparable cell types. We could not perform leukocyte-subset-adjusted or subset-specific sensitivity analyses, which limits the interpretation of the between-sample comparison [22]. Although assay repeatability and plate effects were assessed as described in the Methods, leukocyte subset composition was not measured or adjusted for and may limit the biological comparability of maternal peripheral-blood and neonatal cord-blood TTL estimates [22]. We had no paternal measurement, genetic data or sufficiently detailed information on pregnancy exposures. The subgroup of preterm births and the number of mothers recorded as smokers were small. Because no a priori sample-size calculation was performed, the precision of the primary correlation should be judged from its 95% confidence interval. The sample may have provided limited precision for small associations and exploratory subgroup analyses. The clinical-characteristic analyses were exploratory. After Holm–Bonferroni adjustment across the 16 tests, only the maternal-age association reported in Table 3 remained statistically significant (Holm-adjusted *p* = 0.016). The other nominal findings, including the sex-related associations, did not remain statistically significant after adjustment and should be considered hypothesis-generating rather than confirmatory.

Although 150 of the 167 assessed dyads (89.8%) were included, requiring linked maternal and neonatal samples and complete data may have affected the representativeness of the analytical cohort. The 17 exclusions resulted from withdrawal of consent or incomplete consent documentation, sample-matching problems, an unavailable neonatal sample, or missing essential clinical data. Because excluded and included dyads were not compared on available baseline characteristics, the direction and magnitude of potential selection bias cannot be determined. A prespecified analysis could include paternal telomere length, better measures of maternal exposures, and information on blood-cell composition and laboratory variability. Repeated measurements in children would show whether the relationships observed at birth persist or change [27,28].

## 5. Conclusions

Among the 150 mother–infant dyads, the neonatal assay-derived estimate of total telomere length (TTL) was higher than the maternal estimate in 125 pairs (83.3%). The median neonatal TTL estimate was 1.29 Mb per diploid cell, compared with 0.94 Mb per diploid cell for the maternal estimate. The median within-pair difference between neonatal and maternal TTL estimates was 0.35 Mb per diploid cell. Maternal and neonatal TTL estimates were positively correlated (Spearman ρ = 0.479, *p* < 0.001), although neonatal estimates varied considerably among mothers with similar TTL estimates.

In exploratory adjusted analyses, maternal TTL was positively associated with neonatal TTL (β = 0.662; 95% CI, 0.453 to 0.871). Maternal age was inversely associated with neonatal TTL (β = −0.013 per year; 95% CI, −0.022 to −0.003). Female newborn sex was associated with higher neonatal TTL (β = 0.128; 95% CI, 0.019 to 0.238) and a larger neonatal-minus-maternal difference (β = 0.119; 95% CI, 0.008 to 0.231). These clinical associations are exploratory and require confirmation in larger studies. Prepregnancy BMI, gestational age, and active maternal smoking showed no clear adjusted associations.

## Figures and Tables

**Table 1:** Maternal and neonatal characteristics of the study cohort.

Characteristic	Value
Maternal characteristics	
Maternal age, years	31.4 ± 5.3
Prepregnancy BMI, kg/m^2^	24.7 ± 4.3
Nulliparous before the index delivery *	56 (37.3%)
Active maternal smoking	26 (17.3%)
Chronic hypertension	2 (1.3%)
Gestational hypertension	11 (7.3%)
Preeclampsia	1 (0.7%)
Pre-existing diabetes	1 (0.7%)
Gestational diabetes	7 (4.7%)
Thyroid disease	3 (2.0%)
Anemia	17 (11.3%)
Bronchial asthma	2 (1.3%)
Obstetric characteristics	
Singleton pregnancy	150 (100%)
Vaginal birth	111 (74%)
Cesarean delivery	39 (26%)
Gestational age at delivery, weeks	38.6 ± 1.6
Preterm birth (<37 weeks)	16 (10.7%)
Neonatal characteristics	
Female newborn	76 (50.7%)
Birth weight, g	3198 ± 499
Apgar score at 1 min	8.2 ± 0.7
Apgar score at 5 min	8.9 ± 0.6

Values are presented as mean ± standard deviation or *n* (%), as appropriate. The cohort included 150 mother–infant dyads. BMI, body mass index. Clinical data were complete for all 150 dyads. Maternal age, prepregnancy BMI, parity, smoking status, gestational age, newborn sex and birth weight each had 150 available values and zero missing values. * primiparous at the index delivery.

**Table 2:** Paired maternal and neonatal assay-derived estimates of total telomere length at birth (n = 150 dyads).

Measure	Estimate
Maternal TTL estimate, median (IQR), Mb per diploid cell	0.94 (0.77–1.10)
Neonatal TTL estimate, median (IQR), Mb per diploid cell	1.29 (0.98–1.53)
Within-pair difference in TTL estimates, median (IQR), Mb per diploid cell	0.35 (0.08–0.61); 95% CI, 0.27–0.43
Neonatal TTL estimate higher than maternal TTL estimate	125/150 (83.3%)
Paired Wilcoxon signed-rank test	*p* < 0.001
Spearman correlation between maternal and neonatal TTL estimates	ρ = 0.479; 95% CI, 0.35–0.59; *p* < 0.001

TTL, total telomere length. Values are assay-derived estimates of TTL and are expressed in megabases per diploid cell (Mb per diploid cell). Continuous measurements are presented as median (interquartile range). The within-pair difference was calculated as the neonatal TTL estimate minus the maternal TTL estimate. Paired measurements were compared using the Wilcoxon signed-rank test; their association was assessed using Spearman’s rank correlation coefficient (ρ).

**Table 3:** Exploratory unadjusted associations between clinical characteristics and the neonatal assay-derived estimate of total telomere length (TTL) (n = 150 dyads for each analysis).

Clinical Characteristic	Neonatal TTL by Group, Median (IQR), Mb per Diploid Cell	Association Estimate (Spearman’s ρ or Mean Difference)	95% CI	*p* Value	Holm-Adjusted *p* Value
Maternal age, years	-	ρ = −0.320	−0.457 to −0.168	<0.001	0.016
Prepregnancy BMI, kg/m^2^	-	ρ = −0.180	−0.331 to −0.020	0.028	0.336
Gestational age, weeks	-	ρ = 0.101	−0.060 to 0.257	0.218	1.000
Birth weight, g	-	ρ = 0.119	−0.042 to 0.274	0.147	1.000
Female versus male newborn	Female, n = 76: 1.34 (1.02–1.58) Male, n = 74: 1.21 (0.91–1.47)	+0.128 Mb per diploid cell	+0.016 to +0.240	0.025	0.325
Maternal smoking versus none	Smoking, n = 26: 1.34 (0.97–1.66) No smoking, n = 124: 1.28 (0.98–1.52)	+0.056 Mb per diploid cell	−0.175 to +0.287	0.632	1.000

The outcome was the neonatal assay-derived estimate of TTL. Spearman’s ρ is reported for continuous characteristics. Estimates for newborn sex and maternal smoking represent differences in the neonatal TTL estimate, expressed in Mb per diploid cell, for female versus male newborns and active smoking versus no active smoking, respectively. Reported *p* values are two-sided. For newborn sex and maternal smoking, group-specific values are medians (IQR), whereas estimates are unadjusted mean differences with 95% CIs from Welch’s independent-samples *t*-test. For continuous characteristics, estimates are Spearman’s ρ with 95% CIs.

**Table 4:** Multivariable linear regression model for the neonatal assay-derived estimate of total telomere length (TTL).

Variable	β	95% CI	*p* Value	VIF	Holm-Adjusted *p* Value
Maternal TL (per 1-Mb increase)	+0.662	+0.453 to +0.871	<0.001	1.4	-
Maternal age (per year)	−0.013	−0.022 to −0.003	0.008	1.3	0.120
Prepregnancy BMI (per kg/m^2^)	−0.008	−0.021 to +0.005	0.221	1.5	1.000
Gestational age (per week)	+0.013	−0.018 to +0.044	0.417	1.8	1.000
Female newborn (vs. male)	+0.128	+0.019 to +0.238	0.021	1.1	0.294
Active smoking (vs. no)	+0.032	−0.114 to +0.178	0.665	1.2	1.000
Intercept	+0.700	-	-	-	

The dependent variable was the neonatal assay-derived TTL estimate, expressed in Mb per diploid cell. Regression coefficients (β) represent the adjusted change in the neonatal TTL estimate per unit increase in a continuous predictor, or the adjusted difference between categories for binary predictors. Maternal TTL was expressed in Mb per diploid cell. CI, confidence interval; BMI, body mass index; TTL, total telomere length. The outcome was neonatal telomere length, expressed in Mb per diploid cell. For maternal telomere length, β represents the change in neonatal telomere length (Mb per diploid cell) per 1 Mb per diploid cell increase in maternal telomere length.

**Table 5:** Multivariable linear regression model for the within-pair difference between neonatal and maternal assay-derived estimates of total telomere length (TTL).

Variable	β	95% CI	*p* Value	VIF	Holm-Adjusted *p* Value
Maternal age (per year)	−0.007	−0.015 to +0.001	0.096	1.4	0.960
Prepregnancy BMI (per kg/m^2^)	−0.004	−0.016 to +0.009	0.547	2.2	1.000
Gestational age (per week)	+0.018	−0.013 to +0.049	0.249	1.9	1.000
Female newborn (vs. male)	+0.119	+0.008 to +0.231	0.036	1.1	0.396
Active smoking (vs. no)	+0.053	−0.100 to +0.206	0.495	1.2	1.000
Intercept	−0.100	-	-	-	-

The dependent variable was the neonatal assay-derived TTL estimate minus the maternal assay-derived TTL estimate, expressed in Mb per diploid cell. Regression coefficients (β) represent the adjusted change in this within-pair difference per unit increase in a continuous predictor, or the adjusted difference between categories for binary predictors. CI, confidence interval; BMI, body mass index; TTL, total telomere length.

**Table 6:** Sensitivity analyses including additional clinical covariates.

Additional Covariate	Neonatal TL Model, B (95% CI)	Neonatal–Maternal Difference Model, B (95% CI)
Apgar score at 5 min, per point	+0.012 (−0.018 to +0.042)	+0.009 (−0.021 to +0.039)
Multiparous vs. primiparous at the index delivery	+0.025 (−0.070 to +0.120)	+0.018 (−0.076 to +0.112)
Cesarean vs. vaginal delivery	−0.035 (−0.145 to +0.075)	−0.028 (−0.137 to +0.081)
Any listed maternal condition: yes vs. no	−0.060 (−0.185 to +0.065)	−0.047 (−0.170 to +0.076)

Each additional covariate was added separately to the corresponding primary model; each sensitivity model included 150 dyads. Apgar was coded from 0 to 10; parity as multiparous vs. primiparous; mode of delivery as cesarean vs. vaginal; and maternal condition as any recorded condition vs. none. All estimates are unstandardised coefficients with 95% confidence intervals. Maternal condition was coded as present if at least one of the following was documented: chronic or gestational hypertension, preeclampsia, pre-existing or gestational diabetes, thyroid disease, anemia, or bronchial asthma. The variable was coded as absent when none of these conditions was recorded. Parity as multiparous vs. primiparous at the index delivery, with primiparous as the reference category.

## Data Availability

The data presented in this study are available upon request from the corresponding author. The data are not publicly available due to hospital policy.

## Supplementary Materials

The following supporting information can be downloaded at https://www.mdpi.com/article/10.3390/healthcare14213649/s1, Supplementary File S1: STROBE Reporting Checklist [35]; Supplementary File S2: MIQE 2.0 Reporting Checklist [36].
