# Supplementary material for: Paired Maternal and Neonatal Telomere Length at Birth: An Exploratory Study of Mother–Infant Dyads
Source: Healthcare (Basel). 2026 Oct 28;14(21):3649. doi: 10.3390/healthcare14213649 (PMC13763426; doi:10.3390/healthcare14213649)
Supplement: Supplementary file 1 [file healthcare-14-03649-s001.zip › Supplementary File S1 STROBE Checklist.pdf]

STROBE Reporting Checklist

Completed for the supplied observational manuscript. Checklist selected: combined STROBE checklist for cohort, case-control, and cross-sectional studies; the study reports paired measurements collected around delivery.

Status key: Reported = located in the manuscript; Partial = some information is reported but details remain incomplete; Not reported = not located in the supplied version; N/A = not applicable. Page numbers refer to the supplied manuscript rendering (30 pages).

Item	Reporting item (concise)	Status	Manuscript location / note
1a	Identify the observational design in the title or abstract.	Partial	p. 1; Abstract identifies a single-center observational study, but does not specify a more specific design term.
1b	Summarize methods and findings in a balanced abstract.	Reported	pp. 1–3; Abstract
2	Explain background and scientific rationale.	Reported	pp. 3–4; Introduction
3	State the objectives and any prespecified hypotheses.	Reported	p. 3; final Introduction paragraph
4	Present the main study design early.	Reported	pp. 4–5; Methods, Section 2.1
5	Describe setting, site, and relevant dates.	Reported	pp. 4–5; Section 2.1
6	State eligibility criteria and participant selection.	Reported	pp. 5, 9–10; Sections 2.1 and 3.1 and Figure 1 specify all five exclusion categories and their counts.
7	Define outcomes, exposures, predictors, confounders, and effect modifiers.	Partial	pp. 5–8; Sections 2.2–2.4. Several clinical-variable definitions and ascertainment details remain incomplete.
8	Describe data sources and measurement methods.	Partial	pp. 5–8; Sections 2.2–2.4. qPCR methods are detailed; clinical-variable ascertainment is only partly specified.
9	Describe steps taken to address potential sources of bias.	Partial	pp. 6, 23–24; laboratory blinding and limitations are described, but selection/measurement bias controls are not fully addressed.
10	Explain how study size was determined.	Reported	p. 7; Section 2.4: feasibility/consecutive recruitment; no a priori calculation.
11	Explain treatment of quantitative variables and any cut points.	Partial	pp. 7–8; Section 2.4. Summaries and the preterm cut point are given; handling of all continuous variables is not fully detailed.
12a	Describe statistical methods, including confounding adjustment.	Reported	pp. 7–8; Section 2.4
12b	Describe subgroup, interaction, or sensitivity analyses.	Reported	pp. 7–8 and 18; Section 2.4 describes sensitivity analyses. No subgroup or interaction analyses were performed; additional-covariate sensitivity results are in Table 6.
12c	Explain how missing data were handled.	Reported	p. 7; Section 2.4: all reported clinical variables were complete for the 150 analyzed dyads.
12d	For a cross-sectional study, describe methods accounting for the sampling strategy.	N/A	Not applicable; the study used consecutive single-center recruitment and no complex sampling strategy requiring adjustment.

Item	Reporting item (concise)	Status	Manuscript location / note
12e	Describe sensitivity analyses.	Reported	pp. 7–8 and 18; sensitivity analyses are described in Section 2.4 and reported in Table 6.
13	Report numbers at each study stage and reasons for nonparticipation/exclusion.	Reported	pp. 9–10; Section 3.1 and Figure 1 report the number assessed, the number included, and each exclusion reason with its count.
14	Give participant characteristics and missingness by variable.	Reported	pp. 10–11; Table 1 presents participant characteristics, and its footnote states that clinical data were complete for all 150 dyads.
15	Report outcome data or summary measures.	Reported	pp. 12–15; Results and Tables 2–3
16	Report unadjusted and adjusted estimates with precision and clarify cut points.	Reported	pp. 14–18; Results and Tables 3–5
17	Report other analyses, including sensitivity analyses.	Reported	p. 18; additional-covariate sensitivity analyses are reported in Table 6.
18	Summarize key findings in relation to the objectives.	Reported	pp. 20–25; Discussion and Conclusions
19	Discuss limitations, including direction and likely magnitude of bias.	Partial	pp. 23–24; limitations are discussed, but bias direction/magnitude is not fully assessed.
20	Give a cautious overall interpretation considering objectives, limitations, analyses, and evidence.	Reported	pp. 20–25; Discussion and Conclusions
21	Discuss generalizability.	Partial	pp. 23–24; single-center setting is noted; generalizability could be stated more explicitly.
22	State the source of funding and the funders' role.	Partial	p. 25; publication-cost support is disclosed, but the Funding statement does not specify the funder's role in the study.

Reference: von Elm E, Altman DG, Egger M, et al. The Strengthening the Reporting of Observational Studies in Epidemiology (STROBE) statement: guidelines for reporting observational studies. PLoS Med. 2007;4(10):e296. doi:10.1371/journal.pmed.0040296.
