# Supplementary material for: Paired Maternal and Neonatal Telomere Length at Birth: An Exploratory Study of Mother–Infant Dyads
Source: Healthcare (Basel). 2026 Oct 28;14(21):3649. doi: 10.3390/healthcare14213649 (PMC13763426; doi:10.3390/healthcare14213649)
Supplement: Supplementary file 1 [file healthcare-14-03649-s001.zip › Supplementary File S2 MIQE2 Checklist.pdf]

MIQE 2.0 Reporting Checklist

Completed for the genomic-DNA qPCR telomere assay described in the supplied manuscript. The reverse-transcription section is marked not applicable because no RNA-to-cDNA step was performed.

Status key: Reported = located in the manuscript; Partial = some information is reported but details are absent; Not reported = not located in the supplied manuscript; N/A = not applicable to this genomic-DNA qPCR assay. Page numbers refer to the supplied manuscript rendering (30 pages). Missing details are identified transparently and were not inferred.

MIQE 2.0 section	Reporting item (concise)	Status	Manuscript location / note
1. Reagent preparation	Bioinformatics tools/versions/settings used to design assay	Not reported	Not located; commercial qPCR kit is identified (p. 6, Section 2.3).
1. Reagent preparation	Target gene symbol, species, and sequence accession	Partial	TEL and SCR are named; accession/sequence context is not reported (p. 6, Section 2.3).
1. Reagent preparation	Amplicon location and length	Not reported	-
1. Reagent preparation	Primer/probe sequences or assay context sequences	Not reported	Commercial kit is identified; (p. 6).
1. Reagent preparation	Oligonucleotide modifications and supplier	Partial	Kit supplier is identified; oligonucleotide-specific details are not reported (p. 6).
1. Reagent preparation	Assay optimization details	Not reported	-
2. Sample preparation	Sample types and numbers	Reported	pp. 5–6 and 9; maternal peripheral blood and neonatal cord blood; 150 dyads.
2. Sample preparation	Sampling procedure and time to storage	Reported	p. 6; blood collected at delivery/birth, processed the same day.
2. Sample preparation	Aliquoting, storage conditions, and duration	Partial	p. 6; buffy coat at −80 °C for up to 2 months; DNA at −80 °C for up to 10 months; aliquoting details are not stated.
2. Sample preparation	Extraction method and amount of sample processed	Partial	p. 6; Maxwell kit/instrument identified; starting blood/buffy-coat amount is not specified.
2. Sample preparation	Spike-in nucleic acids and amounts	Not reported	-
2. Sample preparation	Elution volume	Not reported	-
2. Sample preparation	Number of extraction replicates	Not reported	-
2. Sample preparation	Extraction blanks and yield	Partial	p. 6; NTC is reported, but extraction blanks and extraction yield are not reported.
2. Sample preparation	Nucleic-acid quality and quantity methods	Reported	p. 6; fluorometric DNA concentration and A260/A280 and A260/A230 ratios.
2. Sample preparation	DNA storage: temperature, concentration, duration, buffer, aliquots	Partial	p. 6; temperature/duration reported; buffer and aliquot details are not stated.
2. Sample preparation	Template dilution steps	Reported	p. 6; samples diluted to 2 ng/μL.
2. Sample preparation	Template modification and subsequent purification	Not reported	Not reported; the manuscript does not specify whether template modification or post-modification purification was performed.
3. Reverse transcription	RT priming method and concentration	N/A	Genomic DNA qPCR was performed; no reverse-transcription step.

MIQE 2.0 section	Reporting item (concise)	Status	Manuscript location / note
3. Reverse transcription	One-/two-step RT, RNA input, reaction conditions, and RT controls	N/A	Genomic DNA qPCR; no RNA or reverse-transcription step.
3. Reverse transcription	RT reagent identity and cDNA storage	N/A	Genomic DNA qPCR; no cDNA was generated.
4. qPCR protocol	Template treatment before amplification	Reported	p. 6; initial denaturation at 95 °C for 10 min.
4. qPCR protocol	Primer/probe concentration and source	Partial	p. 6; kit and primer-stock volume are reported, but stock/final primer concentrations are not.
4. qPCR protocol	Polymerase, Mg^{2+}, and dNTP identity/concentrations	Partial	p. 6; 2× master mix is identified, but component identities/concentrations are not.
4. qPCR protocol	Buffer/kit manufacturer, catalogue, and lot	Partial	p. 6; kit/manufacturer/catalogue are identified; lot number is not stated.
4. qPCR protocol	Reaction volume and complete cycling conditions	Reported	p. 6; 20-μL reaction composition and cycling program are described.
4. qPCR protocol	qPCR instrument manufacturer and model	Reported	p. 6; ABI 7900HT Real-Time PCR System, Thermo Fisher Scientific.
5. Data analysis	Raw fluorescence data storage/submission using RDML or RDES	Not reported	-
5. Data analysis	Identity and quantification of standards/calibrator	Reported	p. 6; kit-provided reference genomic DNA; assigned value 1.23 ± 0.09 Mb per diploid cell.
5. Data analysis	Baseline correction and Cq determination method	Partial	Cq and ΔΔCq calculations are described (p. 6); baseline/Cq calling settings/software are not specified.
5. Data analysis	qPCR analysis software and version	Not reported	Statistical software is named (p. 7);
5. Data analysis	Positive and negative controls	Reported	p. 6; calibrator and NTC included on each plate.
5. Data analysis	Negative-control frequency and results	Partial	p. 6; NTC included on each plate with no amplification; Cq values are not supplied.
5. Data analysis	Examples/raw results for positive and negative controls	Partial	p. 6; calibrator and NTC behavior are described, but raw example results are not provided.
5. Data analysis	PCR efficiency and estimation method	Partial	p. 6; efficiencies and R^2 are reported; the calculation/standard-curve method is not described.
5. Data analysis	Target-quantity calculation	Reported	p. 6; comparative ΔΔCq formula and conversion to TTL are provided.
5. Data analysis	Biological and technical replicate description	Reported	p. 6; samples analyzed in technical triplicate; paired samples placed in the same plate area.
5. Data analysis	Repeatability/intra-experiment variation	Reported	p. 6; intra-assay CV 3.8%; triplicate acceptance criterion stated.
5. Data analysis	Reproducibility/inter-experiment variation	Reported	p. 6; inter-assay CV 6.2%; plate effects assessed.
5. Data analysis	Limit of detection	Not reported	-
5. Data analysis	Dynamic range/limits of quantification	Partial	p. 6 gives manufacturer-recommended DNA input concentration; analytical assay dynamic range/LOQ is not reported.

MIQE 2.0 section	Reporting item (concise)	Status	Manuscript location / note
5. Data analysis	Validation of reference target (SCR)	Partial	p. 6; SCR is used as single-copy reference; validation/selection rationale is not stated.
5. Data analysis	Normalization/calculation method	Reported	p. 6; TEL and SCR ΔCq values are combined using ΔΔCq relative to the calibrator.
5. Data analysis	Statistical methods	Reported	pp. 7–8; Section 2.4.
5. Data analysis	Significance threshold and statistical power	Partial	pp. 7–8; significance testing is described; no a priori power calculation was performed.
5. Data analysis	Specificity evidence relevant to the assay	Not reported	No assay-specific specificity validation (e.g., amplicon, melt-curve, or sequence evidence) is reported in the supplied manuscript.

Reference: Bustin SA, Ruijter JM, van den Hoff MJB, et al. MIQE 2.0: Revision of the Minimum Information for Publication of Quantitative Real-Time PCR Experiments Guidelines. Clin Chem. 2025;71(6):634–651. doi:10.1093/clinchem/hvaf043.
